# Gene landscape and correlation between B-cell infiltration and programmed death ligand 1 expression in lung adenocarcinoma patients from The Cancer Genome Atlas data set

**DOI:** 10.1371/journal.pone.0208459

**Published:** 2018-12-06

**Authors:** Kuo-Hao Ho, Chih-Ju Chang, Tzu-Wen Huang, Chwen-Ming Shih, Ann-Jeng Liu, Peng-Hsu Chen, Kur-Ta Cheng, Ku-Chung Chen

**Affiliations:** 1 Graduate Institute of Medical Sciences, College of Medicine, Taipei Medical University, Taipei, Taiwan; 2 Department of Biochemistry and Molecular Cell Biology, School of Medicine, College of Medicine, Taipei Medical University, Taipei, Taiwan; 3 Department of Neurosurgery, Cathay General Hospital, Taipei City, Taiwan; 4 Department of Medicine, School of Medicine, Fu Jen Catholic University, New Taipei City, Taiwan; 5 Department of Mechanical Engineering, National Central University, Taoyuan County, Taiwan; 6 Department of Microbiology and Immunology, School of Medicine, College of Medicine, Taipei Medical University, Taipei, Taiwan; 7 Department of Neurosurgery, Taipei City Hospital Ren-Ai Branch, Taipei, Taiwan; University of South Alabama Mitchell Cancer Institute, UNITED STATES

## Abstract

Tumor-infiltrating lymphocytes are related to positive clinical prognoses in numerous cancer types. Programmed death ligand 1 (PD-L1), a mediator of the PD-1 receptor, plays an inhibitory role in cancer immune responses. PD-L1 upregulation can impede infiltrating T-cell functions in lung adenocarcinoma (LUAD), a lung cancer subtype. However, associations between the expression of PD-L1 and infiltration of B cells (a major immunoregulatory cell) remain unknown. Therefore, we investigated the role of infiltrating B cells in LUAD progression and its correlation with PD-L1 expression. The Cancer Genome Atlas (TCGA) LUAD data set was used to explore associations among B-cell infiltration, PD-L1 expression, clinical outcome, and gene landscape. Gene set enrichment analysis was used to explore putative signaling pathways and candidate genes. The drug enrichment analysis was used to identify candidate genes and the related drugs. We found that high B-cell infiltration was correlated with better prognoses; however, PD-L1 may interfere with the survival advantage in patients with high B-cell infiltration. The gene landscape was characterized comprehensively, with distinct PD-L1 levels in cell populations with high B-cell infiltration. We obtained five upregulated signaling pathways from the gene landscape: apoptosis, tumor necrosis factor (TNF)-α signaling via nuclear factor (NF)-κB, apical surface, interferon-α response, and KRAS signaling. Moreover, four candidate genes and their related target drugs were also identified, namely interleukin-2β receptor (*IL2RB*), IL-2γ receptor (*IL2RG*), Toll-like receptor 8 (*TLR8*), and *TNF*. These findings suggest that tumor-infiltrating B cells could act as a clinical factor in anti-PD-L1 immunotherapy for LUAD.

## Introduction

Lymphocyte infiltration into solid tumor tissue is a major prognostic and predictive immunological biomarker of tumor progression. Tumor-infiltrating lymphocytes, such as CD8+ T cells, are associated with cancer prognoses [1]. Moreover, accumulating evidence is suggesting that tumor-infiltrating B cells are strongly associated with positive clinical outcomes in various cancer types [2–5]. In addition to producing antibodies, tumor-infiltrating B cells function as antigen-presenting cells (APCs) to regulate cellular innate immunity in tumor microenvironments. By activating CD8+ T cells, tumor-infiltrating B cells can promote antigen-specific immune responses by inhibiting tumor processes [6]. B cells also produce the opposite effects for tumor immunity and progression. B cells mediate adaptive immunities through the release of circulating cytokines or chemokines to recruit immunosuppressive myeloid cells, resulting in chronic inflammation and de novo carcinogenesis promotion [7]. B cell–derived lymphotoxin can also activate I kappa B kinase (IKK)-α and signal transducer and activator of transcription 3 (STAT3) signaling to promote prostate cancer progression [8]. However, the overall roles and mechanisms of tumor-infiltrating B cells in mediating tumor immunity warrants elucidation.

Immune checkpoints are a diverse group of proteins, including ligands and receptors, that regulate the balance between costimulatory and inhibitory effects on immunity [9]. They not only maintain self-tolerance in preventing autoimmunity formation but also restrict physiological immune cell responses in protecting tissues from damage during pathogenic infection. Cancer cells protect themselves from immune surveillance and antitumor immunity by regulating immune checkpoint expressions [10]. Programmed cell death protein (PD)-1, also known as cluster of differentiation (CD)-279, is a cell surface receptor mainly expressed in activated T, B, and myeloid cells [11]. PD-1 promotes the development of regulatory T cell (Treg), an immunosuppressive T cell type, and subsequently induces anti-inflammatory responses. PD-1 also enhances pathogenic self-reactive T cell apoptosis in lymph nodes [12]. Programmed death ligand 1 (PD-L1), also known as CD274 or B7 homolog 1 (B7-H1), is a specific ligand for PD-1 [13]. High PD-L1 levels are associated with infiltrating T cells and secreted interferon (INF)-γ in the tumor microenvironment; they are also involved in adaptive immune resistance mechanisms [14]. Blockading PD-L1 with an antibody promoted helper T cell (Th) 1 cytokine–activated natural killer cells exhibiting antitumor functions [15], suggesting that PD-1/PD-L1 signaling has critical inhibitory roles in mediating innate and adaptive immunity in the tumor microenvironment.

Human lung cancers, including small-cell lung carcinoma (SCLC) and non-SCLC (NSCLC), are leading causes of malignancy-related mortality worldwide, with fewer than 20% of patients surviving beyond 5 years after diagnosis [16]. Lung adenocarcinoma (LUAD), a main subtype of NSCLC, accounts for approximately 40% of lung cancer patients. LUAD often has a poor clinical outcome and overall 5-year survival. Associations between T-cell infiltration and variant immune checkpoint expressions were described in LUAD progression [17]. T-cell–mediated immune responses and infiltration can significantly delay LUAD malignant tumor processes and could be used to predict poor outcomes in NSCLC [18]. NSCLC patients with high PD-L1 levels have poor prognoses and immature dendritic cell infiltration [19]. PD-L1 expression could be a predictive biomarker in clinical trials testing (neo)adjuvant strategies. Furthermore, in both preclinical and clinical studies PD-1/PD-L1 blockade has achieved robust immune responses and increased survival in NSCLC patients [20]. Further investigation of mechanisms and correlations between PD-L1 and lymphocyte infiltrations may assist future immunotherapy development.

Current immunotherapeutics provides significant benefits of durable remission and prolonged survival in cancer patients, particularly lung cancer patients [21]. Blockading coinhibitory immune checkpoints with monoclonal antibody administration effectively improved T-cell infiltration and function. Atezolizumab and MEDI4736, a newly developed class of drugs for inhibiting PD-L1, effectively attenuated lung cancer progression [20]. However, most studies have focused on the correlation between immune checkpoints and infiltrating T cells. Although tumor-infiltrating B cells participate in mediating tumor immunity, little is known about the associations between PD-L1 and B-cell infiltration in LUAD patients.

Here, we explored the role of B-cell infiltration in LUAD progression using RNA sequencing (RNA-Seq) data on LUAD patients from The Cancer Genome Atlas (TCGA) data set. We evaluated B cell infiltration as a determining factor in anti-PD-L1 immunotherapy response. Furthermore, gene profiles and signaling pathways in different PD-L1 and B-cell infiltration statuses were validated as therapeutic targets for future anti-LUAD drug development.

## Materials and methods

### Obtaining molecular profiles and clinical information of LUAD patients from TCGA

Data on patients (n = 510) with RNA-Seq data and corresponding clinical information (n = 479) were downloaded from the University of California Santa Cruz Cancer Browser (https://genome-cancer.ucsc.edu). The RNA-Seq data were normalized using the RNA-Seq by expectation-maximization method. To obtain LUAD patients’ immune cell infiltration information, an immune infiltration score analyzed using single-sample gene set enrichment analysis (GSEA), with a well-defined reference gene list compiled by Şenbabaoğlu et al. [22], was employed. All data used in this study were derived from the public TCGA database, the use of which does not require approval by an institutional review board or ethics committee.

### Clinical characteristics of B-cell and CD8+ T-cell infiltration

Differences in B-cell and CD8+ T-cell infiltration scores in tumor and paired normal tissues was evaluated using a Wilcoxon signed-rank test. The divergence of these immune infiltration scores in different stages was assessed using Kruskal–Wallis one-way analyses, following Tukey’s method for multiple comparisons. Patients (n = 479) with overall survival information were selected for the survival analysis. Differences in overall survival between various groups were investigated using a log-rank test. Groups that were significantly associated with overall survival were further assessed using a multivariate Cox regression analysis considering patient age, clinical stage, sex, and epidermal growth factor receptor and Kirsten rat sarcoma (KRAS) mutation status.

### Categorization and unsupervised clustering analysis of LUAD patents

The association between PD-L1 gene expression and B-cell infiltration scores was analyzed using multivariate linear regression analysis of the CD8+ T-cell infiltration score. Patients were then divided into four groups based on the median PD-L1 expression and B-cell infiltration score: groups A (low PD-L1/low B-cell infiltration), B (low PD-L1/high B-cell infiltration), C (high PD-L1/high B-cell infiltration), and D (low PD-L1/high B-cell infiltration). To examine discrepancies among these four groups, analysis of variance (ANOVA)-like tests from the edgeR package were used to identify the most variant genes. Patients were hierarchically clustered according to the top 100 genes selected through false discovery rate (FDR). Sample dissimilarity was calculated using the Euclidean method, and a dissimilar matrix was clustered using Ward linkage. The Silhouette algorithm was finally applied to determine the number of clusters was most stable by comparing the scores for each increase in cluster number (up to 10 clusters).

### Identification of pathways and candidate genes for drug discovery

GSEA was performed to compare differences in activated pathways between groups C versus A and groups D versus A. Genes in different groups were ranked according to signal-to-noise ratio. The normalized enrichment score (NES) and FDR were calculated after 1000 permutations. By subtracting the NES between group C versus A and group D versus A, we investigated differences in pathways between these groups. Pathways with an absolute ΔNES of >0.25 were considered to differ.

To further pinpoint genes that sequentially increased in the order of A, D, C, 212 core enrichment genes from pathways with absolute ΔNES values of >0.25 were examined using differential gene expression analysis. In total, 41 genes with sequential 1.5-fold increases in the order of A, D, C (with an FDR of <0.01) were selected for drug discovery.

### Drug target gene enrichment analysis

Manual search of the DrugBank database [23] was used to identify genes targeted by US Food and Drug Administration (FDA)-approved cancer treatment drugs, and 171 target genes were found. To further examine the relation between candidate genes and drug database target genes, we perform fold enrichment analysis with 10^4^ permutations as proposed by Okada et al. [24], we assessed which candidate genes significantly overlapped with the drug target genes. To perform a one-sided permutation test, four genes were first obtained by overlapping 41 differentially expressed and 171 drug target genes. Then, the multiple of enrichment was calculated by dividing four with mean numbers from the null distribution. The *p* value was obtained using a one-sided permutation test.

## Results

### B-cell infiltration more significant in LUAD prognoses than that of CD8+ T cells

**Fig 1:**
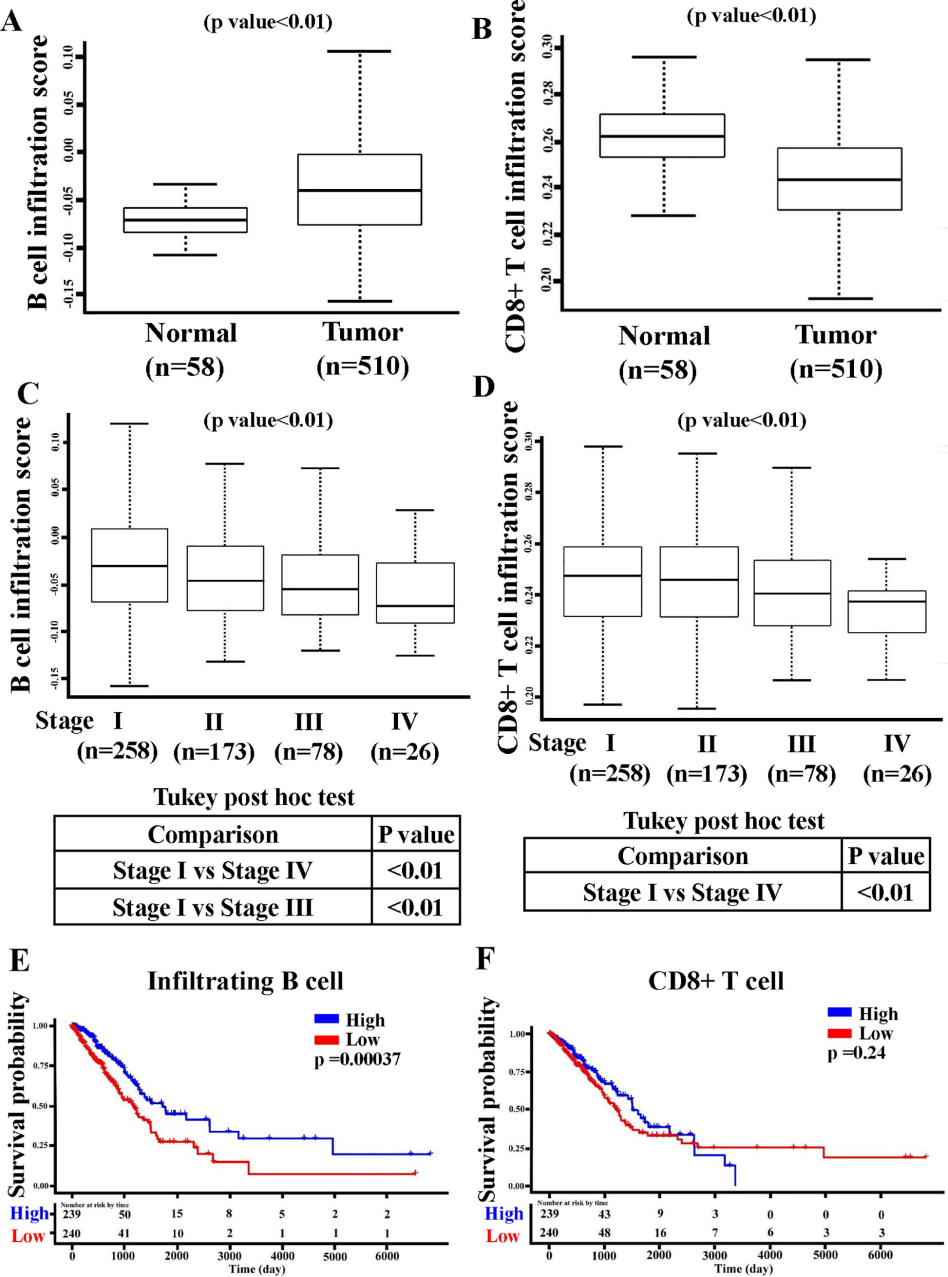
High B-cell infiltration was found in lung adenocarcinoma (LUAD) and was significantly related to patient survival rates. B cell (A) and T cell (B) infiltration scores of LUAD patients (n = 510) and normal group (n = 58) were compared, with the statistical difference calculated using the Wilcoxon rank sum test. B cell (C) and T cell (D) infiltration scores in four different LUAD stages were compared, with the statistical difference calculated using Kruskal–Wallis one-way analysis with Tukey’s post-hoc test. Kaplan–Meier survival curves in LUAD patients with B cell (E) and T cell (F) infiltration scores were analyzed. LUAD patients were divided into two groups based on the median cutoff points for B- and T-cell infiltration scores. The survival rate was measured using a log-rank test.

A study reported that infiltrating CD8+ T cells in lung cancer patients were associated with the histological subtype and degree of dedifferentiation, but not survival [25]. However, no studies have analyzed the role of B-cell infiltration in LUAD prognosis. The demographic and clinical characteristics of LUAD patients from TCGA database are presented in Table 1. In another study, we measured T- and B-cell infiltration scores of LUAD patients [22] and found higher B-cell infiltration scores in LUAD tumor samples (n = 510) than in paired normal tissues (n = 58; Fig 1A). However, CD8+ T-cell infiltration scores were lower in tumor tissues than in paired normal samples (Fig 1B). Previous study has shown that tumor cells, follicular dendritic cells, and T follicular helper cells in lung cancer tissues are able to secrete chemokine, CXCL13, to attract B cells into the tumor tissue [26]. These activated B cells could be transformed into plasma cell to mediate humoral immunity [27]. Beside this, B cell could also activate the T cells to exert its anti-tumor response [28]. In here, we perform gene set enrichment analysis between the high versus low B cell-infiltrated patients, and we also found that humoral immune response and T cell proliferation pathways are also activated (S1 Fig). Hence, high infiltrated B cell in tumor tissues may mediate an ongoing anti-tumor immune response through activation of T cells and antibody-dependent cellular cytotoxicity. On further investigation of the association between infiltration scores and cancer staging, we found that both B- and CD8+ T-cell infiltration scores sequentially decreased from LUAD stage I to IV (Fig 1C and 1D). Moreover, B-cell infiltration scores were significant in both the stage I versus stage IV and stage I versus stage III groups, whereas CD8+ T-cell infiltration scores were only significant in the stage I versus stage IV group. Finally, as shown in Fig 1E, patients with a high B-cell infiltration score had longer survival than patients with a low score (mean survival time difference = 132 days, log-rank test *p* = 0.00037). The variant B-cell infiltration score was also highly significant for patient survival (Table 2). However, no correlation was found between CD8+ T-cell infiltration and survival rate (Fig 1F), suggesting that B-cell infiltration may play a greater role in LUAD prognosis than does CD8+ T-cell infiltration.

**Table 1:** Clinical characteristics of lung adenocarcinoma patients.

Data	TCGA
**Number of patients**	479
**Age (years), mean (SD)**	65.3 (9.9)
**Female, No. (%)**	348 (52.1)
**Median survivor** **follow-up (days)**	468 days
** Smoking history, No. (%)**	
Yes	396 (79.7)
No	69 (14.4)
Unknown	14 (2.9)
** Stage, No. (%)**	
I	258 (53.9)
II	173 (36.2)
III	78 (16.3)
IV	26 (5.4)
**EGFR gene variation**	
Non-mutation	181 (37.8)
Mutation	28 (5.8)
Unknown	270 (56.4)
**KRAS gene variation**	
Non-mutation	147 (30.7)
Mutation	62 (12.9)
Unknown	270 (56.4)

TCGA, The Cancer Genome Atlas; SD, standard deviation; EGFR, epidermal growth factor receptor; KRAS, Kirsten rat sarcoma.

**Table 2:** Univariate and multivariate Cox regression analyses of risk factors for overall survival.

Variable	Univariate Cox regression		Multivariate Cox regression	
	Hazard ratio	*p* value	Hazard ratio	*p* value
**B cell infiltration**				
**High vs. Low**	**0.54**	**<0.001**	**0.44**	**<0.001**
**Age (years)**				
Aged>65 vs. Age≤65	1.18	0.33	-	-
**Gender**				
Female vs. Male	0.96	0.83	-	-
**Stage**				
Stage II vs. Stage I	**2.32**	**<0.001**	**2.13**	**<0.001**
Stage III vs. Stage I	**4.24**	**<0.001**	**3.74**	**<0.001**
Stage IV vs. Stage I	**3.08**	**<0.001**	1.65	0.340
**EGFR gene variation**				
Mutation vs. non-mutation	**2.15**	**0.01**	1.73	0.090
**KRAS gene variation**				
Mutation vs. non-mutation	0.90	0.71	-	-

EGFR, epidermal growth factor receptor; KRAS, Kirsten rat sarcoma.

### Correlation between PD-L1 levels and B-cell infiltration

**Fig 2:**
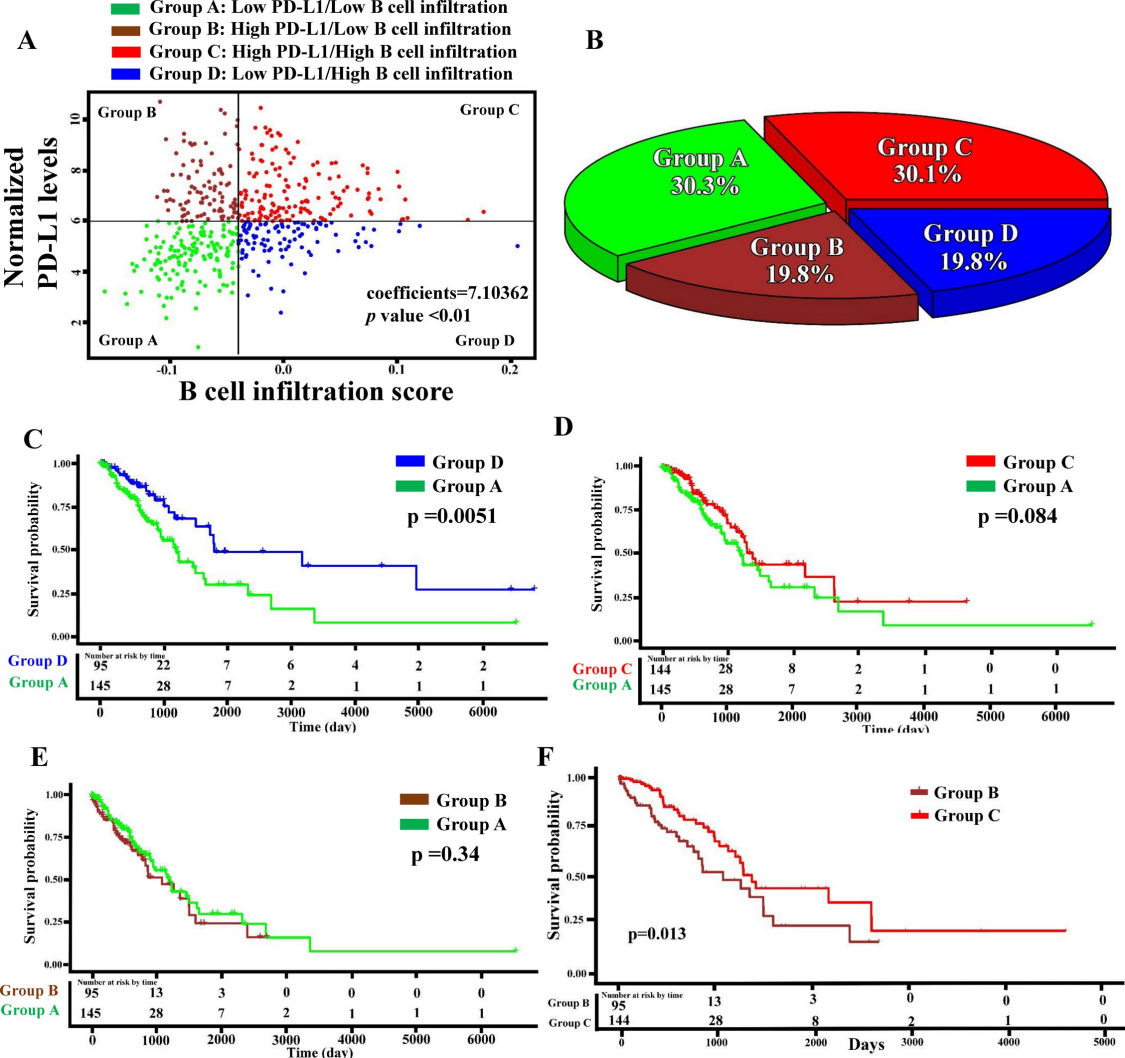
Programmed death ligand 1 (PD-L1) expression levels and B-cell infiltration were associated with patient survival rates. (A) Positive correlation between normalized PD-L1 levels and B-cell infiltration scores, measured using multivariate linear regression analyses. Different PD-L1 and B-cell infiltration levels were defined for groups A to D. (B) The pie chart shows the distribution of patient numbers (%) among the four groups. Kaplan–Meier survival curves were analyzed for lung adenocarcinoma (LUAD) patients in group D versus group A (C), group C versus group A (D), group B versus group A (E), and group B versus group C (F). The survival rate was calculated using a log-rank test.

High PD-L1 expression in lung cancer patients was associated with histological types and overall survival and contributed to poor prognosis and tumor cell immune escape [19]. However, the association between PD-L1 expression and B-cell infiltration remains unclear. By analyzing B-cell infiltration scores with normalized PD-L1 levels from the RNA-Seq data of LUAD patients in TCGA database, a significant and positive correlation between PD-L1 expression and B-cell infiltration was identified (n = 479, coefficient = 7.10362, *p* < 0.01; Fig 2A). Further considering CD8+ T-cell infiltration as a covariate, a significantly positive correlation between B-cell infiltration and PD-L1 levels was still found (infiltrating B cells: coefficient = 5.09 and *p* < 0.01; infiltrating CD8 T cells: coefficient = 11.56 and *p* < 0.01), suggesting that the correlation of B-cell infiltration with PD-L1 levels was independent of CD8 T-cell infiltration. LUAD patients were then divided into four groups based on the median cutoff points for B cell scores and normalized PD-L1 levels (Fig 2B), namely groups A (low PD-L1/low B-cell infiltration, n = 145, 30.3% of patients), B (high PD-L1/low B-cell infiltration, n = 95, 19.8% of patients), C (high PD-L1/high B-cell infiltration, n = 144, 30.1% of patients), and D (low PD-L1/high B-cell infiltration, n = 95, 19.8% of patients). Since group A had immunological ignorance, we defined it as the basal control group. Compared with this group (Fig 2C–2F), only group D patients were found to have significantly higher survival rates (mean survival time difference = 239 days, log-rank test *p* = 0.0051). This finding was also confirmed by univariate and multivariate Cox regression analyses (Table 3). Although group C patients also had high B-cell infiltration, their high PD-L1 expression levels resulted in poor survival rates. All the results indicated that greater B-cell infiltration could be related to high PD-L1 expression. PD-L1 acted as a negative regulator to influence B-cell infiltration–mediated tumor immunity.

**Table 3:** Univariate and multivariate Cox regression analyses of risk factors for overall survival in low PD-L1/high B cell infiltration and low PD-L1/low B cell infiltration patients (*n* = 240).

Variable	Univariate cox regression		Multivariate cox regression	
	Hazard Ratio	P value	Hazard Ratio	P value
**B cell infiltration**				
**High vs Low**	**0.54**	**<0.001**	**0.44**	**<0.001**
**PD-L1**				
**High vs Low**	**0.858**	0.37	-	-
**Age**				
Age>65 vs Age< = 65	1.18	0.33	-	-
**Sex**				
Female vs Male	0.96	0.83	-	-
**Stage**				
Stage II vs Stage I	**2.32**	**<0.001**	**2.13**	**<0.001**
Stage III vs Stage I	**4.24**	**<0.001**	**3.74**	**<0.001**
Stage IV vs Stage I	**3.08**	**<0.001**	1.65	0.340
**EGFR gene variation**				
Mutation vs non-mutation	**2.15**	**0.01**	1.73	0.090
**KRAS gene variation**				
Mutation vs non-mutation	0.90	0.71	-	-

EGFR, epidermal growth factor receptor; KRAS, Kirsten rat sarcoma.

### Characterization of gene signatures and signaling dissimilarities with different PD-L1 expression and B-cell infiltration

**Fig 3:**
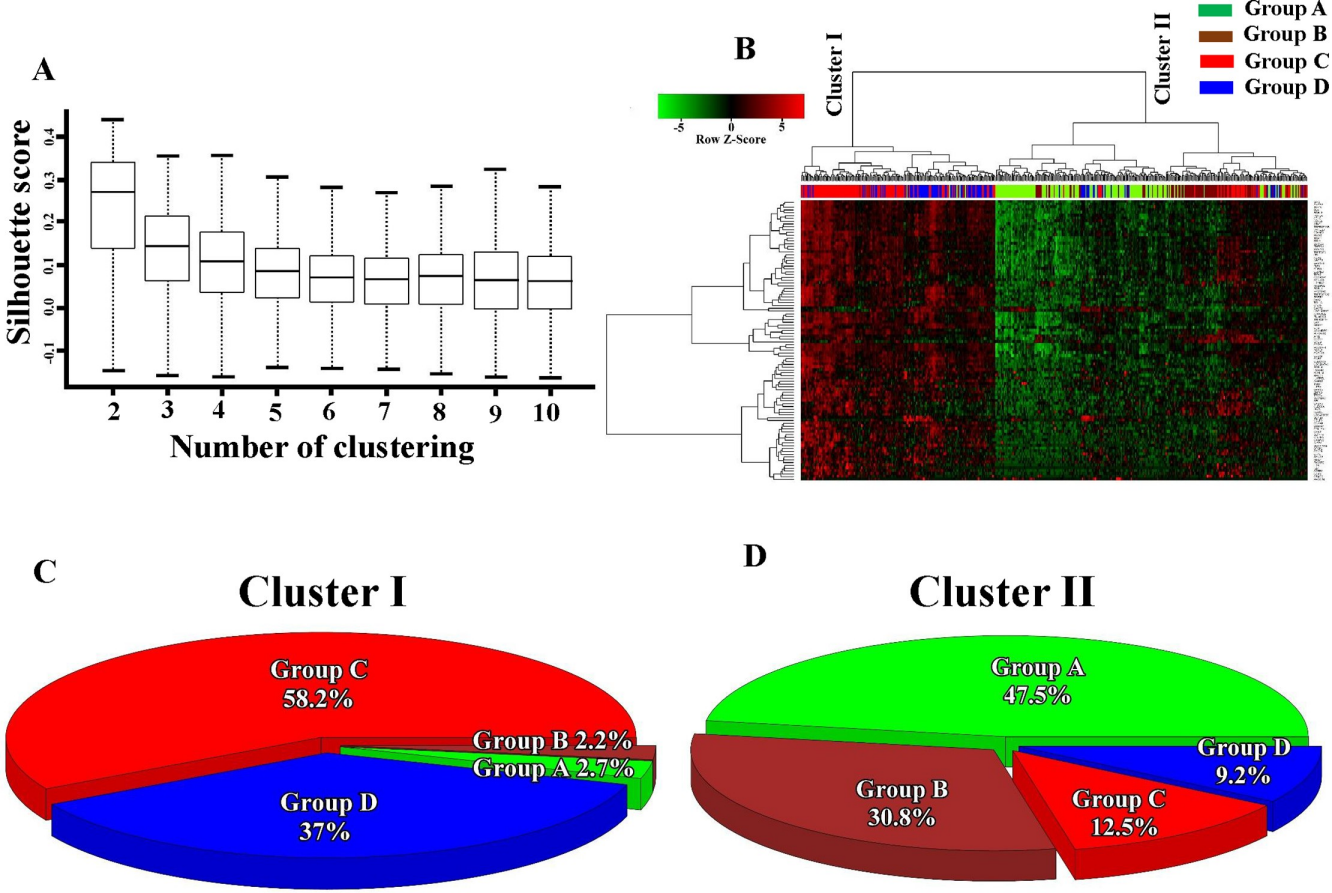
Gene signature dissimilarity among four groups with different PD-L1 and B-cell infiltration levels. (A) Silhouette score with different numbers of clusters, calculated using cluster robustness analyses. (B) Heat map with the top 100 differentially expressed genes (DEGs) in the four groups. DEGs with a significant false discovery rate were analyzed using ANOVA-like tests, after which unsupervised hierarchical cluster analysis was performed. Stable clusters I and II were generated. The pie chart shows distributions of patients (%) from the four groups in clusters I (C) and II (D).

To explore discrepancies in molecular levels related to PD-L1 expression and B-cell infiltration, we first investigated the diversity of gene signatures among the four groups with different PD-L1 levels and B-cell infiltration. The top 100 significantly differentially expressed genes within the four groups were selected (S1 File). We found that two clusters, namely clusters I and II, showed the greatest robustness (Fig 3A and 3B). On examining the distribution of patients in clusters I and II (%), we found that groups C and D, both with high B-cell infiltration but different PD-L1 levels, contained most cluster-I patients (95.2%; Fig 3A). By contrast, cluster II mainly consisted of patients from groups A and B (78.3%), both with low B-cell infiltration and different PD-L1 levels (Fig 3B). Because this study focused only on the immunosuppressive role of PD-L1 in highly B cell–infiltrated tumor tissues, group B patients (high PD-L1 expression and low B-cell infiltration) were excluded from subsequent analyses.

**Fig 4:**
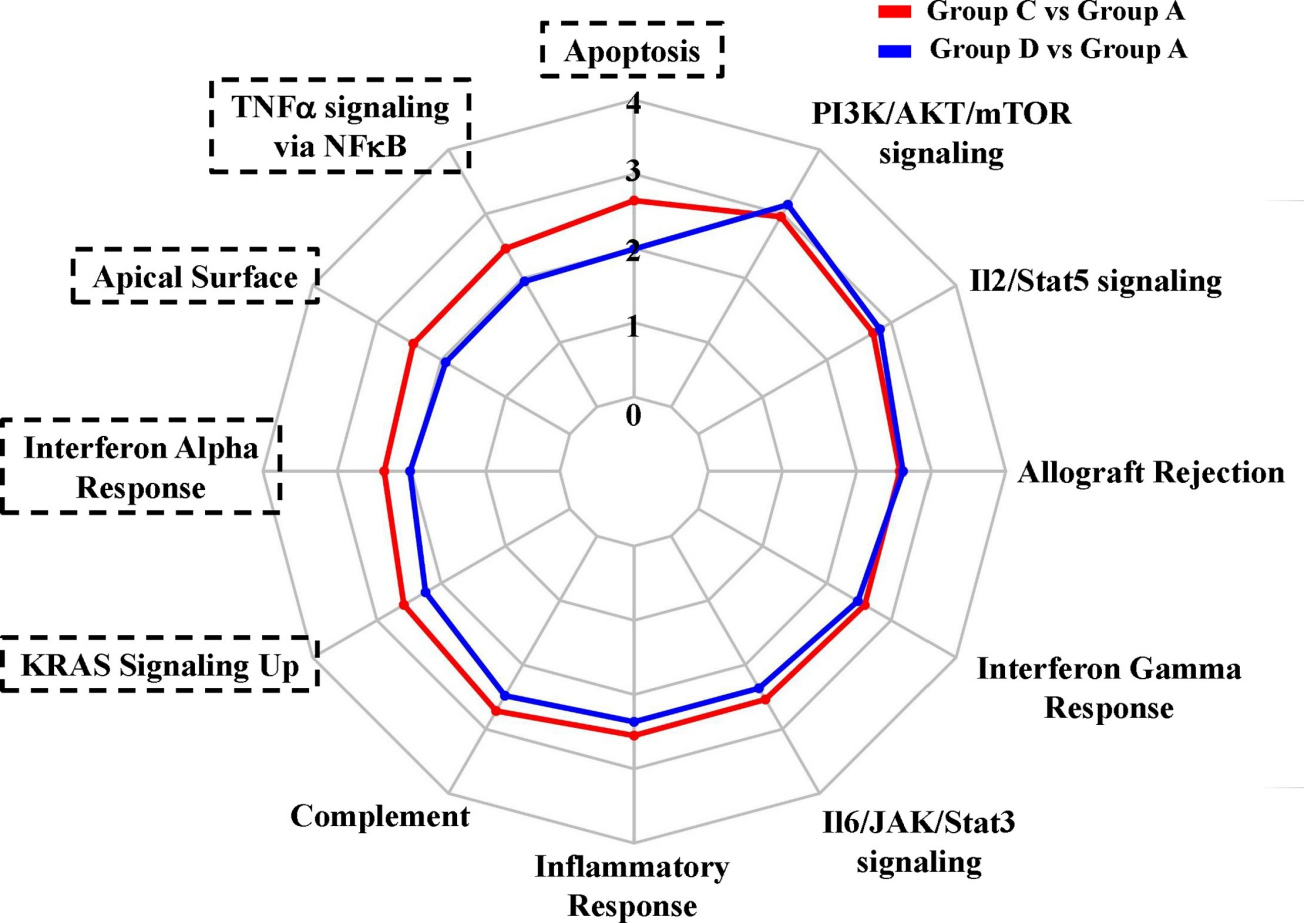
**Radar chart displaying normalized enrichment scores of group C versus group A (red) and group D versus group A (blue) in hallmark pathways.** Hallmark pathways more overrepresented in group C versus group A than in group D versus group A are highlighted in black brackets.

High B-cell infiltration with low PD-L1 was significantly related with longer survival in LUAD patients (group D versus A). However, high B-cell–infiltration patients with high PD-L1 had no survival benefit compared with low B-cell–infiltration/low-PD-L1 expression patients (group C versus A). To distinguish the effects of PD-L1 in groups with high B-cell infiltration, the divergence of gene functions between groups C and D was analyzed (Table 4). Twelve enrichment pathways were activated in group C versus A (FDR ≤ 0.05). On further comparing the activated pathways of group D versus A, five additional overrepresented pathways were selected (ΔNES ≥ 0.25) in group C versus A, namely apoptosis, TNF-α signaling via NF-κB, apical surface, INF-α response, and KRAS signaling (Fig 4). The results demonstrated that these signaling pathways were activated in the presence of B-cell infiltration without PD-L1. Moreover, patients with B-cell infiltration and elevated PD-L1 expression exhibited higher levels of these pathways. In general, varying B-cell infiltration and PD-L1 status resulted in dissimilar gene and signaling signatures in LUAD patients.

**Table 4:** Enriched pathways associated with different PD-L1/B cell infiltration statuses.

HALLMARK gene set	Group C vs. Group A		Group D vs. Group A		Δ NES
	NES	FDR	NES	FDR	
**Apoptosis**	**1.98**	**0.02**	**1.49**	**0.18**	**0.49**
**TNF-α signaling via NF-κB**	**1.85**	**0.02**	**1.46**	**0.19**	**0.38**
**Apical surface**	**1.83**	**0.03**	**1.45**	**0.19**	**0.38**
**Interferon-α response**	**1.77**	**0.03**	**1.51**	**0.19**	**0.26**
**KRAS signaling upregulation**	**1.94**	**0.02**	**1.68**	**0.09**	**0.25**
Complement	2.04	0.01	1.86	0.04	0.18
Inflammatory response	1.91	0.02	1.77	0.06	0.14
Il6/JAK/STAT3 signaling	1.90	0.02	1.77	0.05	0.13
Interferon-γ response	1.94	0.02	1.86	0.04	0.08
Allograft rejection	1.93	0.02	1.97	0.02	-0.03
IL-2/STAT5 signaling	2.04	0.02	2.12	0.01	-0.08
PI3K/AKT/mTOR signaling	2.22	0.01	2.36	<0.01	-0.14

NES, normalized enrichment score; FDR, false discovery rate; delta NES, NES (group C-group D); TNF, tumor necrosis factor; NF, nuclear factor; KRAS, Kirsten rat sarcoma; JAK, Janus kinase; STAT, signal transducer and activator of transcription; IL, interleukin; PI3K, phosphoinositide 3-kinase; AKT, protein kinase B; mTOR, mammalian target of rapamycin.

### Investigation of drug-targeting genes related to B-cell infiltration and PD-L1

**Fig 5:**
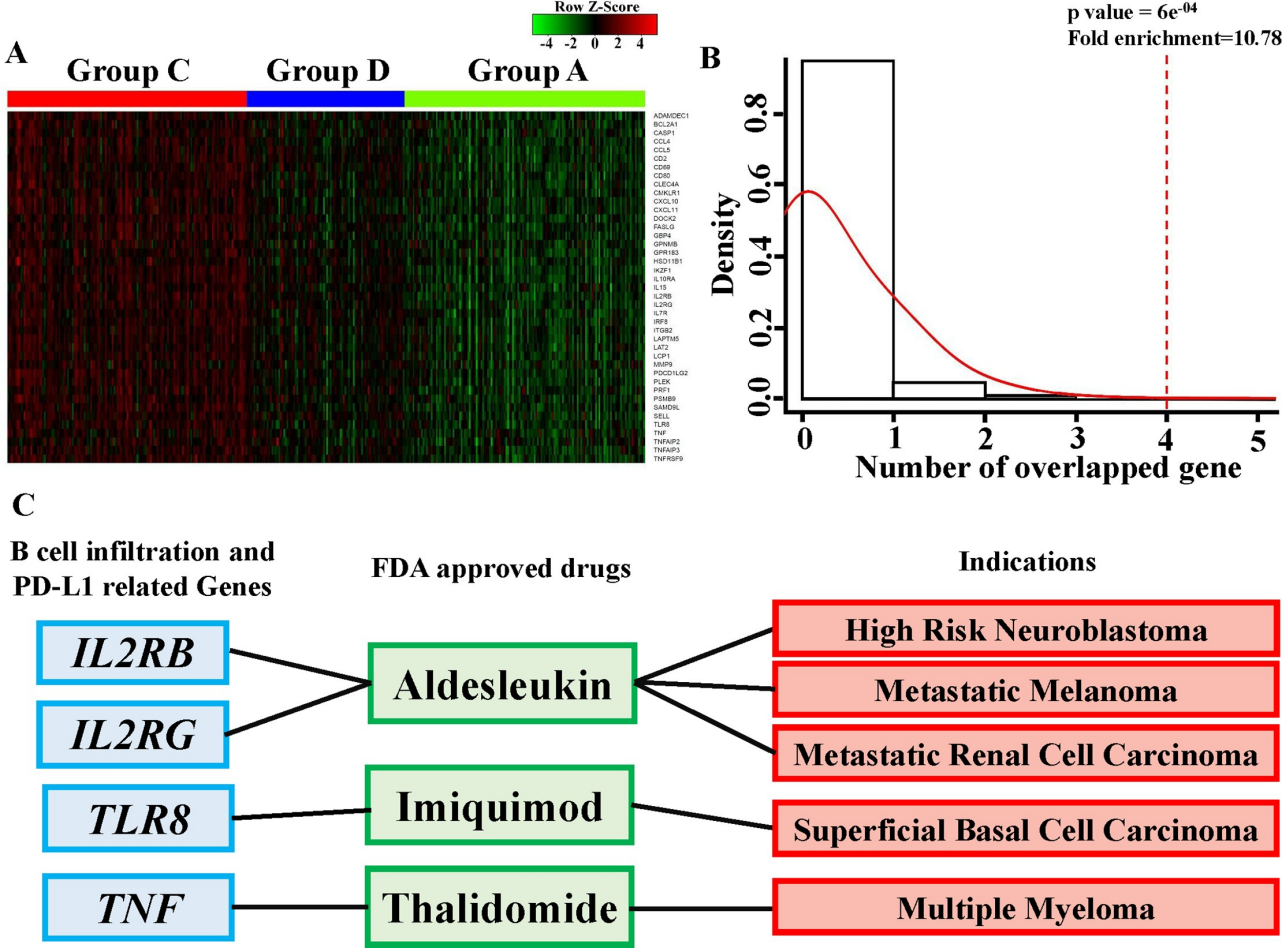
Identification of drug target genes related to B-cell infiltration and PD-L1. (A) Heat map showing differentially expressed genes (DEGs) in group C versus group A compared with group D versus group A. A total of 212 core enrichment genes from the five pathways in Fig 4 were analyzed. A total of 41 DEGs with a significant false discovery rate (<0.01) and sequential 1.5-fold increase in the order of A, D, C were selected. (B) Results of one-sided permutation analyses. The null distribution was developed through the intersection of 171 drug target genes cancer treatment–related from DrugBank and 41 genes randomly selected from 19,035 protein-coding genes with 10^4^ permutations. The dotted line indicates the number of genes associated with B-cell infiltration and PD-L1 expression that also had therapeutic potential for cancer treatment (10.78-fold enrichment, *p* = 6e^-04^). Four overlapping genes from the 41 DEGs and 171 drug target genes were obtained through drug enrichment analysis. (C) Scheme of relationships of genes related to B-cell infiltration and PD-L1 to approved drugs and indications.

To further identify differentially expressed genes (DEGs) among these five significantly increased signaling pathways in group C versus A, compared with group D versus A, changes in expressions of 212 core enrichment genes from these pathways were analyzed. We identified 41 significant DEGs (FDR < 0.01) and sequentially increased 1.5-fold in the order of A, D, C (Fig 5A and S1 Table). Finally, we evaluated the potential role in drug discovery of high B-cell infiltration–and PD-L1–related genes. The 171 drug target genes from DrugBank [23] (S2 Table) corresponding to drugs approved for cancer treatment demonstrated a significant overlap with the 41 DEGs related to B-cell infiltration and PD-L1; that is, four genes overlapped (10.78-fold enrichment by a permutation analysis, *p* = 6e^-04^; Fig 5B), namely interleukin 2β receptor (*IL2RB*), interleukin 2γ receptor (*IL2RG*), Toll-like receptor 8 (*TLR8*), and *TNF* (Fig 5C). Three approved cancer therapy drugs, namely aldesleukin, imiquimod, and thalidomide, were identified through this analysis. Therefore, these B-cell infiltration and PD-L1–related genes may possess a critical potential in drug development for LUAD patients.

## Discussion

The concepts of cancer immunoediting, comprising cancer immunosurveillance, equilibrium, and escape, are increasingly prominent in clinical cancer research and therapy [29, 30]. Cancer immunoediting protects a host against carcinogenesis, but may also enhance tumor evasion from immune destruction. Variation in the levels of infiltrating lymphocytes, immunocheckpoints, and some microenvironmental cytokines are crucial roles in deciding the fate of tumors. Further understanding associations among these cancer immunity-related factors during tumor progression can help in correctly classifying patients for the optimal combined treatment for each tumor type. Both tumor-infiltrating B cells and PD-L1 immunocheckpoints are involved in lung cancer immunity [17, 31]. Little is known about the roles and interrelations of B-cell infiltration and PD-L1 expression in lung cancer, particularly LUAD. In this study, we found that B-cell infiltration levels were higher in LUAD tumor samples than in paired normal tissues from TCGA by comparing B-cell infiltration scores, which were significantly associated with tumor progression and patient survival. A positive correlation between B-cell infiltration and PD-L1 expressions was thus verified. Dissimilar gene signatures and signaling pathways were also identified in patients with high B-cell infiltration with or without elevation in PD-L1 expression. Finally, drug target genes and the corresponding FDA-approved drugs for patients with high B-cell infiltration or high PD-L1 status were investigated. Our findings described a possible landscape of B-cell infiltration and PD-L1 expression within LUAD tumors, thus indicating a valuable novel direction for therapy and drug development.

Blockading immunecheckpoints such as the PD-1/PD-L1 complex demonstrated remarkable clinical benefits with a long immunogenic response in the tumor microenvironment, thus leading to a paradigm shift in cancer therapies. However, in NSCLC patients, humanized anti-PD-L1 antibody treatment led to a relatively lower objective response rate, higher 3- and 4-grade adverse effect rates, and higher number of drug-related deaths compared with chemotherapy or tyrosine kinase inhibitor (TKI) therapy [32]. This suggested that not all populations are suitable for anti-PD-L1 therapy. Moreover, Herbst et al. [33] reported that anti-PD-L1 therapy produced significant responses, particularly in cancer patients with high levels of PD-L1 and infiltrating immune cells—suggesting that the degree of immune cell infiltration may be related to the efficacy of anti-PD-L1 therapy in lung cancer patients. In this study, we found a positive correlation between PD-L1 levels and B-cell infiltration score. Patients with high B-cell infiltration and low PD-L1 level had a better survival rate than those with high B-cell infiltration and high PD-L1 level. Our findings indicated that elevated PD-L1 expression may impede B-cell infiltration–mediated immune responses in LUAD patients, resulting in poor prognosis. Moreover, B-cell infiltration may represent a crucial clinical factor for future anti-PD-L1 therapies in lung cancer patients.

According to the stratification rule developed by Teng et al. [34], we divided LUAD patients into four groups (Fig 2). By using unsupervised hierarchical cluster analyses (Fig 3), we found that patients in groups C (high PD-L1/high B-cell infiltration) and D (low PD-L1/high B-cell infiltration) had similar activated gene signature patterns but different patient survival rates. Thus, these genes may be activated during B-cell infiltration but may not demonstrate sufficient inhibitory effects against the infiltrating B-cell–promoted immune system. However, elevated PD-L1 levels accompanied by higher activated gene expressions resulted in even greater immune dysfunction in the presence of B-cell infiltration. To further investigate the molecular mechanisms, we identified five more highly activated signaling pathways between the two populations, groups C and D (Fig 4). A study reported that PD-L1 upregulation promotes CD8+ T cell apoptosis [35]. Moreover, activation of the B-cell antigen receptor can induce growth arrest and apoptosis of germinal center B cells [36]. TNF-α was identified as an autocrine growth factor from B cells, which upregulated PD-L1 expression through NF-κB signaling activation [37]. IFN-α can induce the amplification of naïve B-cell activation and PD-L1-mediated T-cell apoptosis [38]. KRAS is critical in B-cell lymphopoiesis and is necessary for enhanced PD-L1 expression [39]. All these studies suggest that the overactivated signaling pathways in our findings are critical for functions exhibiting B-cell infiltration and enhanced PD-L1. Finally, our results indicated that four genes (*IL2RB*, *IL2RG*, *TLR8*, and *TNF*) and their related target drugs, including aldesleukin (recombinant IL-2), imiquimod (Toll-like receptor activator), and thalidomide (immunomodulatory drug) may provide replacement strategies for developing adjuvant agents in combined anti-PD-L1 cancer immunotherapy.

This study has some limitations. Because the TCGA lists a limited number of factors, many crucial factors, such as performance status, weight loss, anemia, and treatment schedule, could not be considered in the overall survival analyses. A significant imbalance in the number of groups studied also existed in this study. Because of the lack of publicly accessible histology data, we could not revalidate our results. In addition, the dual protumor and antitumor effects of B cells remain unclear. Several B-cell subtypes, such as plasma cells, memory B cells, B1a (Ly-1 B) cells, and B2 cells, are in circulation. Regulatory B cells (Bregs), a newly identified B-cell subtype, play a suppressive role in regulating inflammation in autoimmune diseases. However, the B-cell infiltration score used in this study could not distinguish these B-cell subtypes. Precisely defining the actual role of B cells infiltration in LUAD progression was also difficult. Identifying specific surface markers or developing new techniques of classifying infiltrating B cell subtypes would assist in understanding B-cell–mediated immune mechanisms and promoting cancer immunotherapy research.

With the clinical success of immunotherapies, further understanding the roles and mechanisms of immunoinflammatory factors in the tumor microenvironment is of major significance for next-generation cancer therapies. In this study, we presented the evidence of gene correlations, dissimilar gene signatures, and variant signaling pathways in LUAD patients with differing B-cell infiltration and PD-L1 status. We also indicated some therapeutic target genes and related FDA-approved drugs that may be used in future drug development strategies and combined treatments with anti-PD-L1 drugs for lung cancer. In summary, improved characterization of the functions and mechanisms of tumor-infiltrating B cells and PD-L1 not only improves knowledge of tumor immune mechanisms but also suggests potential directions for future drug development and cancer immunotherapies.

## Supporting information

S1 Fig Enrichment signaling pathways are positively correlated with high B cell infiltration. (A) Humoral immune response (B) T cell proliferation.
(PDF)

S1 File List of top 100 significantly differentially expressed genes within the four groups of different PD-L1 levels and B-cell infiltration. (XLSX)

S1 Table The list of all differential expressed genes. The 41 genes in bold are sequential upregulations.
(PDF)

S2 Table The list of drug target genes related to cancer treatment. (PDF)

## Abbreviations

APC: antigen-presenting cell
Breg: regulatory B cell
FDR: false discovery rate
IKK: kappa B kinase
IL2RB: interleukin 2β receptor
IL2RG: interleukin 2γ receptor
KRAS: Kirsten rat sarcoma
NSCLC: non-small-cell lung carcinoma
PD: programmed cell death
PD-L1: programmed death ligand 1
LUAD: lung adenocarcinoma
NES: normalized enrichment score
SCLC: small-cell lung carcinoma
STAT: signal transducer and activator of transcription
TCGA: The Cancer Genome Atlas
Th: T helper cell
hTLR8: Toll-like receptor 8
TNF: tumor necrosis factor
Treg: regulatory T cell
