# Supplementary material for: Gene landscape and correlation between B-cell infiltration and programmed death ligand 1 expression in lung adenocarcinoma patients from The Cancer Genome Atlas data set
Source: PLoS One. 2018 Dec 6;13(12):e0208459. doi: 10.1371/journal.pone.0208459 (PMC6283571; doi:10.1371/journal.pone.0208459)
Supplement: S1 Fig — (A) Humoral immune response (B) T cell proliferation. (PDF) [file pone.0208459.s001.pdf]

S1 Fig

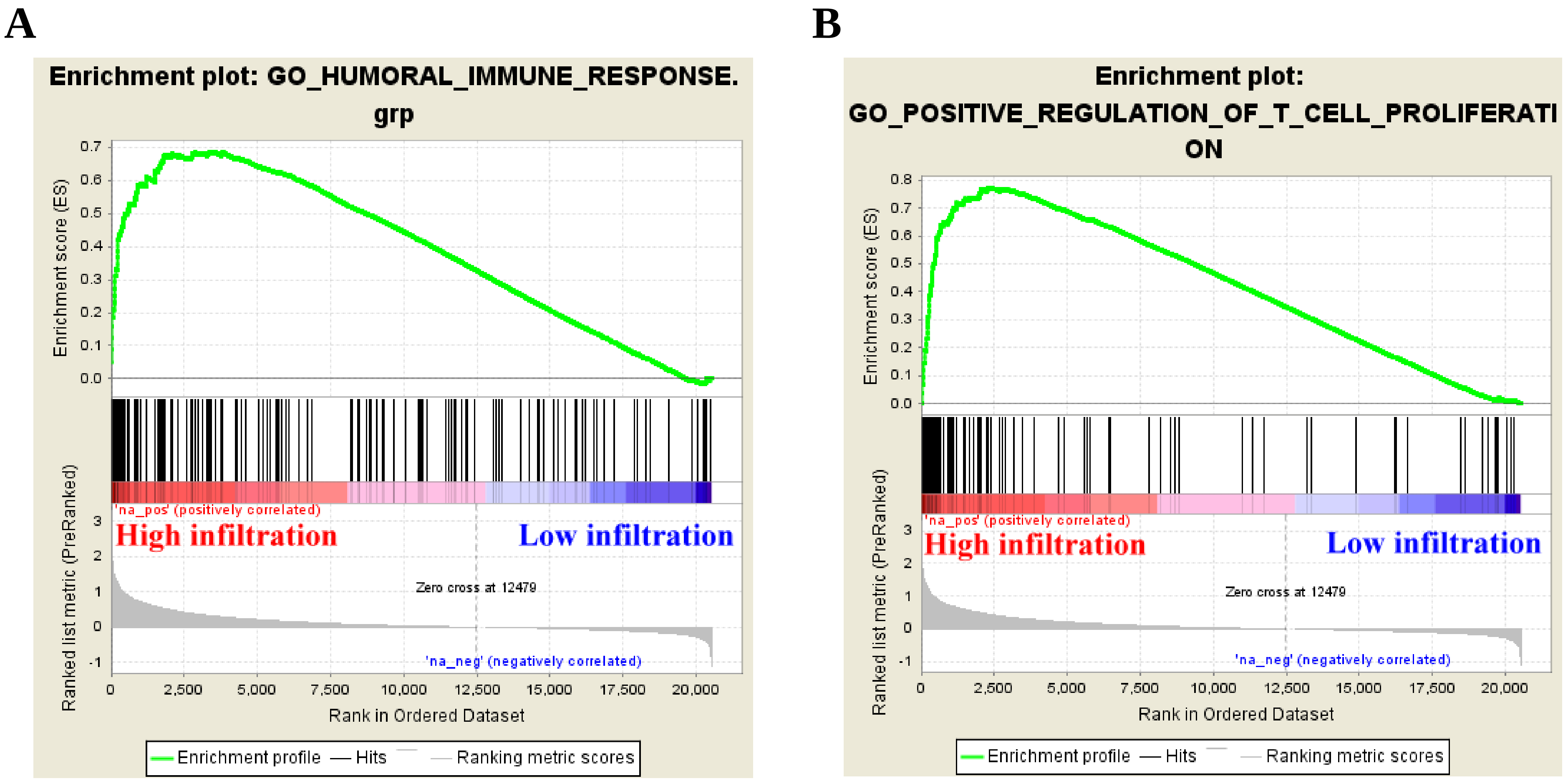

S1 Figure. Enrichment signaling pathways are positively correlated with high B cell infiltration. (A) Humoral immune response (B) T cell proliferation.
