# Supplementary material for: Gene landscape and correlation between B-cell infiltration and programmed death ligand 1 expression in lung adenocarcinoma patients from The Cancer Genome Atlas data set
Source: PLoS One. 2018 Dec 6;13(12):e0208459. doi: 10.1371/journal.pone.0208459 (PMC6283571; doi:10.1371/journal.pone.0208459)
Supplement: S1 Table — The 41 genes in bold are sequential upregulations. (PDF) [file pone.0208459.s003.pdf]

S1 Table. The list of all differential expressed genes. The 41 genes in bold are sequential upregulations.

Gene	Group D vs Group A		Group C vs Group D	
	Log 2 fold change	FDR	Log 2 fold change	FDR
**ADAMDEC1**	1.52	1.15E-09	1.34	7.01E-08
**CD2**	1.40	5.49E-21	0.69	1.25E-06
**SELL**	1.39	5.11E-16	0.59	0.000461
**TNFRSF9**	1.38	5.36E-15	1.08	7.02E-10
**IL7R**	1.32	2.69E-11	0.94	1.25E-06
**IKZF1**	1.29	4.46E-23	0.65	1.42E-07
**IL2RG**	1.24	2.42E-17	0.61	1.77E-05
**CCL5**	1.22	1.04E-13	0.82	3.24E-07
**CD69**	1.20	7.15E-12	0.59	0.000588
**IRF8**	1.18	5.63E-17	0.68	7.57E-07
**IL2RB**	1.15	3.20E-17	0.87	8.91E-11
**FASLG**	1.13	1.15E-10	1.02	4.92E-09
**IL10RA**	1.06	3.10E-18	0.72	1.33E-09
**DOCK2**	1.05	7.90E-14	0.85	1.1E-09
**GPR183**	1.00	1.85E-11	0.61	2.97E-05
**PLEK**	0.97	4.58E-12	0.98	3.57E-12
**LCP1**	0.88	2.14E-11	0.78	2.58E-09
**PRF1**	0.86	1.10E-07	0.78	8.42E-07
**CD80**	0.84	5.89E-09	1.00	5.82E-12
**MMP9**	0.83	0.000149	0.81	0.000249
**PDCD1LG2**	0.82	1.25E-08	1.29	1.76E-17
**CXCL10**	0.82	7.24E-05	1.59	1.22E-13
**PSMB9**	0.80	2.93E-09	0.78	6.74E-09
**TLR8**	0.80	7.54E-06	1.31	1.07E-12
**HSD11B1**	0.79	4.38E-07	0.97	7.6E-10
**CXCL11**	0.77	0.000304	1.63	3.28E-13
**CCL4**	0.75	2.78E-07	1.10	3.41E-13
**CLEC4A**	0.75	3.29E-07	0.86	4.28E-09
**ITGB2**	0.75	1.67E-06	0.93	3.27E-09
**TNF**	0.72	0.000217	0.75	0.000113
**IL15**	0.70	3.64E-08	0.62	9.33E-07
**GBP4**	0.70	4.80E-07	1.17	6.36E-16

**TNFAIP3**	0.69	1.47E-08	0.64	1.43E-07
**LAPTM5**	0.69	5.80E-08	0.77	1.08E-09
**LAT2**	0.68	1.61E-07	0.62	1.22E-06
**CASP1**	0.63	4.69E-07	0.67	1.13E-07
**GPNMB**	0.63	0.000167	0.76	5.79E-06
**SAMD9L**	0.63	1.94E-06	1.09	1.7E-15
**BCL2A1**	0.62	7.39E-05	0.85	5.78E-08
**TNFAIP2**	0.59	0.000249	0.62	0.000129
CMKLR1	0.59	4.49E-05	1.05	1.79E-12
MAP4K1	1.52	3.35E-25	0.42	0.002446
NR0B2	1.36	0.000304	-1.17	0.001872
CXCR4	0.98	3.48E-15	0.30	0.013992
CD37	1.22	8.96E-18	0.57	3.82E-05
ABCB1	1.13	1.90E-11	0.12	0.506212
CD74	0.98	1.75E-09	0.47	0.003228
LEF1	0.83	5.88E-10	-0.02	0.918451
DUSP2	0.85	1.12E-09	0.40	0.003314
PRDM1	0.80	1.61E-10	0.41	0.000862
SNAP91	1.20	6.53E-08	-0.45	0.04303
MSC	0.78	1.13E-06	0.53	0.000999
TRAF1	0.73	2.94E-11	0.52	1.85E-06
CCND2	0.72	1.74E-07	0.44	0.001394
TNFAIP8	0.71	1.82E-10	0.51	4.16E-06
KIF5C	1.20	2.99E-07	-0.45	0.057748
RTP4	0.70	1.18E-05	0.49	0.001966
GATA3	0.70	2.04E-05	0.51	0.001966
SLC2A6	0.70	4.38E-07	0.45	0.001079
CD83	0.65	3.05E-07	0.48	0.000152
PDE4B	0.64	1.21E-06	0.46	0.000458
IL7	0.64	6.34E-05	0.51	0.001393
GYPC	0.63	2.28E-07	0.38	0.001924
LY96	0.62	2.76E-06	0.19	0.158256
GHRL	0.78	2.97E-06	0.23	0.190817
PLCB2	0.72	7.80E-06	0.18	0.282367
CD38	0.88	1.46E-05	0.44	0.033113
TNC	0.62	0.012764	0.69	0.005425
ID2	0.57	1.94E-05	0.03	0.861672

GADD45G	0.59	0.001562	-0.72	0.000141
MAP3K1	0.36	2.79E-05	0.09	0.323392
MAL	0.90	3.23E-05	0.18	0.406961
BTG2	0.65	3.60E-05	-0.16	0.323392
ST6GAL1	0.51	3.66E-05	-0.04	0.786946
IRF1	0.58	2.70E-08	0.73	5.82E-12
HLA-C	0.58	8.30E-07	0.48	3.62E-05
ICOSLG	0.44	6.89E-05	0.16	0.144545
EGR3	0.57	0.0022	0.52	0.005013
PTGER4	0.56	0.000101	0.77	1.32E-07
ETS1	0.56	2.05E-07	0.53	8.29E-07
PSME1	0.31	0.00011	0.08	0.323307
CTSS	0.56	1.94E-06	0.65	4.06E-08
UBE2L6	0.56	2.05E-07	0.56	1.43E-07
C3AR1	0.55	0.000146	1.03	5.06E-12
F13A1	0.55	0.010573	0.87	5.52E-05
HDAC9	0.53	0.005019	0.64	0.000767
WARS	0.53	5.86E-06	0.76	1.53E-10
CCRL2	0.52	0.000555	0.51	0.000835
TPH1	0.68	0.000175	-0.27	0.149356
CD160	0.46	0.000211	0.29	0.020566
PCP4	1.63	0.000216	-0.55	0.229613
B2M	0.52	7.32E-07	0.55	1.66E-07
FCER1G	0.51	0.000129	0.91	2.41E-11
SLC16A6	0.49	0.001178	0.73	1.2E-06
TNFRSF1B	0.48	1.47E-05	0.50	7.77E-06
PSME2	0.32	0.000381	0.24	0.010096
TAP1	0.48	3.55E-05	0.84	2.87E-12
HGF	0.48	0.016623	0.60	0.002944
ALDH1A3	0.65	0.000485	0.44	0.020916
IRF9	0.30	0.000542	0.14	0.12806
BIRC3	0.48	0.005139	0.89	2.24E-07
PIGR	1.46	0.000759	-0.51	0.254785
TLR2	0.58	0.000913	0.44	0.012242
OLR1	0.48	0.014815	0.98	6.76E-07
IFI44L	0.46	0.033052	0.91	4.78E-05
IL12B	0.55	0.001301	0.30	0.089783

CASP7	0.24	0.001395	0.10	0.202355
GFPT2	0.46	0.00412	0.65	4.93E-05
TUBB2A	0.60	0.001626	-0.01	0.972824
LPPR4	0.56	0.001626	0.04	0.861672
AKAP7	0.37	0.001665	0.19	0.115079
IFITM1	0.53	0.001703	-0.01	0.978035
TMEM176B	0.51	0.00189	0.26	0.125136
IL15RA	0.46	2.53E-05	0.73	4.72E-11
GCH1	0.35	0.002237	0.19	0.11326
NAP1L2	0.63	0.002662	-0.03	0.911471
DCN	0.48	0.003497	0.35	0.035868
TRIB2	0.39	0.003987	0.08	0.563877
MAFB	0.45	0.000145	0.79	6.26E-11
UBA7	0.44	0.000118	0.33	0.004304
IFI30	0.44	0.000149	0.60	2.26E-07
IL1B	0.44	0.015849	1.06	9.63E-09
EPB41L3	0.44	0.005195	0.61	9.07E-05
SLC2A4	0.44	0.00538	-0.51	0.001329
CSF2RA	0.41	0.017884	0.84	1.5E-06
SNAP25	0.67	0.005199	-0.23	0.373516
EGR2	0.40	0.010747	0.78	8.54E-07
CYLD	0.40	2.78E-07	0.32	2.77E-05
IGFBP6	0.58	0.006505	0.09	0.709196
IL33	0.54	0.006572	0.28	0.183581
SAMD9	0.39	0.005195	0.86	1.36E-09
MDGA1	0.39	0.025258	0.53	0.003013
CSF1	0.38	0.006829	0.69	1.4E-06
ITGBL1	0.48	0.008675	-0.02	0.91454
TXNIP	0.37	0.009469	0.23	0.117101
GAS1	0.49	0.00964	0.39	0.044609
CX3CL1	0.38	0.067555	0.66	0.00254
GADD45A	0.35	0.010747	0.31	0.028638
MMP2	0.38	0.017884	0.50	0.002073
SULF2	0.40	0.012428	0.33	0.04186
PSMB8	0.38	0.000404	0.36	0.000809
CD14	0.37	0.007834	0.87	1.64E-09
CDC25B	0.31	0.013714	-0.02	0.8894

CD44	0.36	0.005542	0.54	2.98E-05
BATF2	0.35	0.010747	0.82	3.27E-09
LUM	0.39	0.016324	0.35	0.037796
TMEM176A	0.42	0.016419	0.16	0.376314
FAS	0.34	0.013	0.79	1.42E-08
RGS16	0.38	0.016659	0.20	0.221759
ALDH1A2	0.72	0.01679	-0.03	0.925821
SCG3	0.79	0.017084	-0.22	0.541195
PTX3	0.34	0.055679	0.58	0.001703
PRRX1	0.36	0.017884	0.38	0.015721
AFAP1L2	0.33	0.041955	0.45	0.007063
CCNA1	0.65	0.017884	0.59	0.039452
HMGB2	0.26	0.019008	0.00	0.978035
IFI44	0.32	0.06808	0.71	0.000111
VWA5A	0.34	0.021174	-0.13	0.397644
SATB1	0.34	0.022747	-0.17	0.282367
CSF2	0.56	0.024012	0.55	0.032199
IL6	0.32	0.157195	0.84	0.000474
EPSTI1	0.32	0.024399	0.98	3E-11
SMAD7	0.22	0.026048	0.03	0.777442
GPRC5B	0.34	0.026217	0.06	0.753338
CCL20	0.71	0.026547	0.09	0.808829
PEG3	0.39	0.030176	0.16	0.402151
APOD	0.63	0.030592	-0.51	0.096164
TMEM8B	0.23	0.03235	-0.23	0.043531
LPAR6	0.32	0.005199	0.35	0.002086
IL23A	0.38	0.037241	0.31	0.112142
SOD2	0.30	0.007381	0.60	1.26E-07
CXCL6	0.67	0.042908	0.59	0.094855
CFB	0.42	0.045371	0.33	0.146258
ISG20	0.26	0.048862	-0.11	0.418364
CLU	0.46	0.049279	-0.54	0.027113
PCSK1N	0.67	0.049461	-0.60	0.098506
FGF9	0.56	0.050058	-0.70	0.018974
SCUBE1	0.44	0.05098	0.37	0.115079
TSPAN7	0.45	0.051254	-0.42	0.089783
ENO2	0.29	0.051683	0.07	0.661707

SERPINA3	0.64	0.053398	-0.19	0.618021
LAMP3	0.40	0.053414	0.28	0.206624
CROCC	0.25	0.055226	-0.21	0.129646
TRIM21	0.29	0.000479	0.28	0.000802
G0S2	0.29	0.121087	0.61	0.00176
ETV1	0.33	0.058864	0.20	0.28802
AIM1	0.26	0.091082	1.04	3.33E-10
DDX60	0.26	0.058013	0.80	1.04E-08
THY1	0.28	0.067555	0.18	0.265389
LAP3	0.25	0.001059	0.55	5.06E-12
IFI27	0.40	0.069629	0.32	0.182009
BST2	0.27	0.084981	0.36	0.027068
PMAIP1	0.24	0.165682	0.56	0.002446
TGFBR3	0.27	0.099586	0.16	0.384057
HOXD11	0.44	0.103434	-0.42	0.148835
DDIT3	0.20	0.107262	-0.30	0.023127
DPYD	0.23	0.120037	0.83	1.7E-07
HERC6	0.23	0.067555	0.42	0.001692
TMEM140	0.21	0.005019	0.54	5.94E-12
TRIM14	0.21	0.020438	0.57	8.35E-10
NGF	0.37	0.175565	-0.01	0.982425
RTN4RL1	0.35	0.187173	-0.04	0.911471
FEZ1	0.16	0.193435	0.22	0.099071
IFNB1	0.10	0.198909	0.07	0.36172
TGFB2	0.23	0.255802	0.29	0.189599
OASL	0.19	0.27951	0.80	2.8E-05
RSAD2	0.17	0.267602	0.84	3.14E-07
