# Supplementary material for: Gene landscape and correlation between B-cell infiltration and programmed death ligand 1 expression in lung adenocarcinoma patients from The Cancer Genome Atlas data set
Source: PLoS One. 2018 Dec 6;13(12):e0208459. doi: 10.1371/journal.pone.0208459 (PMC6283571; doi:10.1371/journal.pone.0208459)
Supplement: S2 Table — (PDF) [file pone.0208459.s004.pdf]

**S2 Table. The list of drug target genes related to cancer treatment**

| | | | | |
|---|---|---|---|---|
| ABL1 | DDR2 | HDAC2 | NR3C1 | RRM2B |
| ACPP | DHFR | HDAC3 | NTRK1 | RXRA |
| AKT1 | DNMT1 | HDAC4 | ORM1 | RXRB |
| ALK | EBP | HDAC6 | ORM2 | RXRG |
| ALPPL2 | EGFR | HDAC8 | PARP1 | SH2B3 |
| APEX1 | ELANE | IKBKB | PARP2 | SLC6A2 |
| AR | ENPP1 | IL2RA | PARP3 | SLC6A3 |
| ATIC | EPHA2 | IL2RB | PDCD1 | SLC6A4 |
| BCL2 | ERBB2 | IL2RG | PDGFRA | SRC |
| BCR | ERBB4 | ITK | PDGFRB | STMN4 |
| BRAF | ESR1 | JAK1 | PGF | TEK |
| BTK | ESR2 | JAK2 | PGR | TLR7 |
| C1QA | ESRRG | JAK3 | PNP | TLR8 |
| C1QB | FCGR1A | JUN | POLA1 | TNF |
| C1QC | FCGR2A | KDR | POLE | TNFSF11 |
| C1R | FCGR2B | KIT | POLE2 | TOP1 |
| C1S | FCGR2C | LCK | POLE3 | TOP1MT |
| CAMK2G | FCGR3A | LDLR | POLE4 | TOP2A |
| CCND1 | FCGR3B | LHCGR | PRKCA | TOP2B |
| CD274 | FDPS | LYN | PRKCB | TUBA1A |
| CD33 | FGF1 | MAOA | PRKCD | TUBA4A |
| CD38 | FGFR1 | MAOB | PRKCE | TUBB |
| CDK2 | FGFR2 | MAP2 | PRKCG | TUBB1 |
| CDK4 | FGFR3 | MAP2K1 | PRKCI | TUBB3 |
| CDK6 | FGFR4 | MAP2K2 | PRKCQ | TUBD1 |
| CHD1 | FLT1 | MAP3K2 | PRKCZ | TUBE1 |
| CMPK1 | FLT3 | MAP4 | PRLR | TUBG1 |
| CRBN | FLT4 | MAPK1 | PSMB1 | TXNRD1 |
| CSF1R | FPGS | MAPK11 | PSMB5 | TYMS |
| CSF3R | FRK | MAPK3 | PTGS2 | VEGFA |
| CTLA4 | GART | MAPT | PTK6 | VEGFB |
| CYP17A1 | GGPS1 | MET | RAF1 | |
| CYP19A1 | GNRHR | MTOR | RET | |
| DCK | HCK | NFKB1 | RRM1 | |
